# Comparative Prognostic Value of the Triglyceride‐Glucose Index, hs‐CRP, and the Derived C‐Reactive Protein–Triglyceride‐Glucose Index for Major Adverse Cardiovascular and Cerebrovascular Events in Acute Coronary Syndrome: Insights From a Prospective Cohort

**DOI:** 10.1002/mco2.71130

**Published:** 2026-11-13

**Authors:** Wenjie Wang, Xiangfei Meng, Yang Zhang, Li Lin, Zehao Zhao, Jiayi Han, Ruixiang Feng, Yingxuan Bai, Liwei Guo, Yaodong Ding, Yong Zeng

**Affiliations:** ^1^ Department of Cardiology Beijing Anzhen Hospital Capital Medical University Beijing People's Republic of China

**Keywords:** acute coronary syndrome (ACS), C‐reactive protein‐triglyceride‐glucose index (CTI), high‐sensitivity C‐reactive protein (hs‐CRP), major adverse cardiovascular and cerebrovascular events (MACCE), triglyceride‐glucose index (TyG)

## Abstract

The C‐reactive protein–triglyceride‐glucose index (CTI) combines inflammation with metabolic status, but its prognostic value in patients with acute coronary syndrome (ACS) remains unclear. We studied 9131 patients with ACS admitted between 2021 and 2024 to examine the associations of CTI, the triglyceride‐glucose (TyG) index, and high‐sensitivity C‐reactive protein (hs‐CRP) with long‐term major adverse cardiovascular and cerebrovascular events (MACCE). Cox regression models, restricted cubic spline analyses, and predictive performance analyses were performed. After a median follow‐up of 707 days, 801 patients developed MACCE. In fully adjusted models, CTI (hazard ratio [HR] = 1.248, 95% CI: 1.150–1.355), the TyG index (HR = 1.235, 95% CI: 1.115–1.367), and hs‐CRP (HR = 1.031, 95% CI: 1.018–1.047) were all significantly associated with MACCE risk. Patients in the highest CTI quartile had a greater risk of MACCE than those in the lowest quartile (HR = 1.651, 95% CI: 1.333–2.045). Restricted cubic spline analyses showed positive associations without significant nonlinearity. CTI yielded a numerically higher area under the curve (AUC) than the TyG index and hs‐CRP, although the improvement was modest. These findings suggest that CTI may provide supplementary metabolic‐inflammatory information for MACCE risk assessment in ACS, but external validation is needed.

## Graphical abstract

The TyG index, hs‐CRP, and CTI were associated with long‐term MACCE risk in ACS patients, with CTI showing numerically higher predictive performance. Higher CTI indicated increased MACCE risk.

•Why did we undertake this study?

To improve long‐term MACCE risk assessment in patients with ACS.

•What specific question(s) do we wanted to answer?

Can CTI predict long‐term MACCE risk in patients with ACS?

•What did we find?

CTI was independently associated with MACCE and showed modest incremental predictive value.

•What are the implications of our findings?

CTI may provide adjunctive metabolic‐inflammatory information for MACCE risk assessment following ACS.

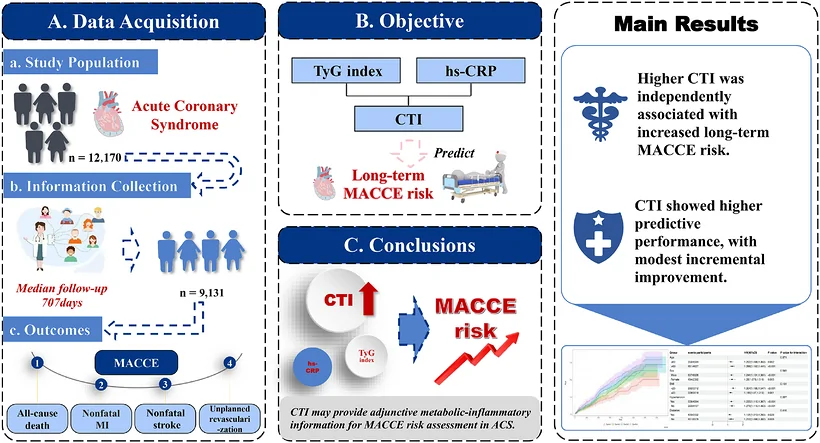

## 1 Introduction

Worldwide, the burden of acute coronary syndrome (ACS) increases markedly with age in both men and women [1]. An estimated 7 million new cases of ACS are diagnosed worldwide each year [2]. A previous registry‐based study reported that the average cost of the initial hospitalization for patients with ACS was $18,931 [3]. Beyond direct medical expenses, ACS can also lead to severe clinical consequences, such as heart failure, malignant arrhythmias, and reinfarction, which substantially increase patients’ long‐term health risks and economic burden and may pose a persistent threat to their lives [4, 5, 6]. In current clinical practice, ST‐segment changes on electrocardiography and high‐sensitivity cardiac troponin (hs‐cTn) testing remain central to the diagnosis, early risk assessment, and treatment planning of ACS [7]. Even so, tools used in the acute phase do not fully address the need for medium‐ and long‐term prognostic assessment after ACS, highlighting the value of readily obtainable markers for follow‐up risk evaluation. Existing biomarkers may have limited predictive value for major adverse cardiovascular and cerebrovascular events (MACCE), such as death and recurrent myocardial infarction, underscoring the need to explore additional markers for risk stratification and prognostic management [1].

Insulin resistance (IR) reflects an impaired biological response to insulin in peripheral tissues and is often accompanied by metabolic abnormalities, such as hyperglycemia, hypertension, and dyslipidemia, all of which are associated with adverse cardiovascular outcomes [8, 9, 10]. The reference method for determining insulin sensitivity, the hyperinsulinemic‐euglycemic clamp, is nevertheless constrained by its technical complexity and high cost, limiting its applicability in routine clinical practice [11]. In this context, the triglyceride‐glucose (TyG) index, conveniently computed using fasting triglyceride and glucose (Glu) values, has been extensively employed as a practical surrogate marker of IR. In an adult Korean cohort, individuals exhibiting an elevated TyG index demonstrated more pronounced progression of coronary artery calcification, which reflects atherosclerotic plaque burden in the coronary arteries [12]. Among patients with ACS, the TyG index has also been reported to provide prognostic information regarding subsequent adverse cardiovascular events, with this association persisting even after adjustment for diabetes status [8, 13].

Inflammatory activation contributes substantially to the progression of ACS [14]. Pro‐inflammatory cytokines, including interleukin‐1 and tumor necrosis factor‐α, can promote endothelial injury, plaque destabilization, and myocardial damage, thereby contributing to adverse clinical outcomes following ACS [15, 16]. High‐sensitivity C‐reactive protein (hs‐CRP) is a readily available indicator of systemic inflammatory activity that is routinely used in clinical practice. Higher hs‐CRP concentrations have been associated with atherosclerotic progression, plaque instability, and a greater likelihood of cardiovascular events among patients with ACS [17, 18].

Given the involvement of both metabolic disturbances and inflammation in ACS, the C‐reactive protein–triglyceride‐glucose index (CTI) has been introduced as an integrated measure of inflammatory status and IR, integrating hs‐CRP and the TyG index [1, 19, 20]. Since its introduction by Ruan et al. [19], CTI has been investigated in various clinical populations. Findings from earlier studies show that higher CTI values correlate with greater stroke risk among individuals with hypertension [21] and with higher cardiovascular disease incidence and mortality [20]. Atherosclerosis, the underlying pathological basis of ACS, is closely related to both metabolic disorders, including IR‐related lipid abnormalities, and inflammatory responses throughout plaque formation, progression, and rupture [22, 23]. Therefore, CTI may capture the combined metabolic‐inflammatory burden and provide additional prognostic information for cardiovascular risk assessment.

Nevertheless, data on CTI in patients with ACS remain limited, particularly regarding long‐term MACCE. It also remains unclear whether CTI provides prognostic information beyond that offered by either the TyG index or hs‐CRP used separately. To address these questions, we analyzed data from 9131 patients with ACS treated at Beijing Anzhen Hospital between 2021 and 2024. We investigated the associations of CTI, the TyG index, and hs‐CRP with long‐term MACCE, compared their prognostic performance, and assessed whether the association between CTI and MACCE varied across major clinical subgroups.

## 2 Results

### 2.1 Baseline Characteristics According to CTI Quartiles

A total of 9131 patients contributed to the final analytic cohort, as shown in Figure 1. Their baseline profiles, stratified by CTI quartile, are summarized in Table 1. Overall, 6828 patients (74.8%) were male. Across increasing CTI quartiles, patients tended to be slightly younger, had a higher body mass index (BMI), and exhibited a greater probability of having hypertension. The use of glucose‐lowering and antihypertensive therapies was also more frequent in the higher CTI quartiles, and the distribution of ACS subtypes varied across quartiles. Regarding laboratory parameters, increasing CTI quartiles corresponded to higher values for glucose, triglycerides, the TyG index, hs‐CRP, HbA1c, hs‐TnI, creatinine (Cr), urea, uric acid, total cholesterol (TC), and low‐density lipoprotein cholesterol (LDL‐C), whereas high‐density lipoprotein cholesterol (HDL‐C) levels decreased. MACCE occurred in 801 patients during a follow‐up period with a median of 707 days. Follow‐up outcomes stratified by CTI quartile are detailed in Table S1, whereas baseline profiles categorized by MACCE occurrence are summarized in Table S2. Compared with patients who did not experience MACCE, those who developed MACCE were of greater age, had hypertension more often, and showed higher levels of the TyG index, hs‐CRP, and CTI.

**FIGURE 1:**
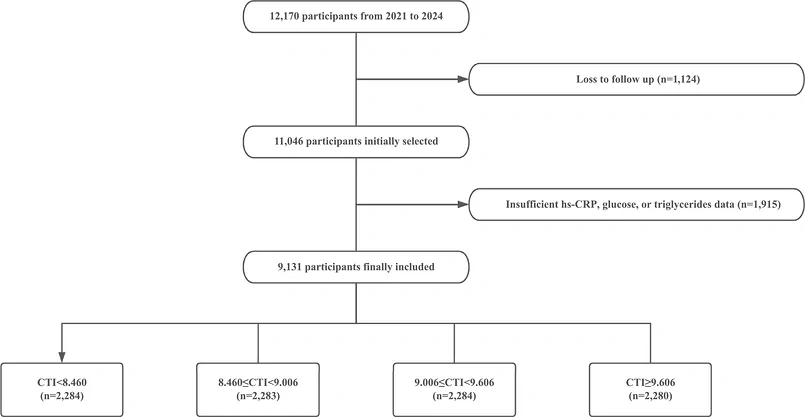
Flowchart of participant selection.

**TABLE 1:** Baseline characteristics of the study population according to CTI quartiles.

Characteristics	Overall (*n* = 9131)	Quartile 1 (< 8.460; *n* = 2284)	Quartile 2 (8.460–9.006; *n* = 2283)	Quartile 3 (9.006–9.606, *n* = 2284)	Quartile 4 (≥ 9.606, *n* = 2280)	*p* value
Age, years	61.00 [54.00, 67.00]	61.00 [55.00, 67.00]	61.00 [55.00, 67.00]	60.00 [53.00, 67.00]	60.00 [52.00, 67.00]	< 0.001
Male, *n* (%)	6828 (74.8)	1741 (76.2)	1713 (75.0)	1701 (74.5)	1673 (73.4)	0.162
BMI, kg/m^2^	25.91 [23.89, 28.06]	24.91 [22.98, 26.96]	25.77 [23.74, 27.78]	26.30 [24.24, 28.40]	26.64 [24.68, 28.98]	< 0.001
Hypertension, *n* (%)	4994 (54.7)	1119 (49.0)	1206 (52.8)	1290 (56.5)	1379 (60.5)	< 0.001
Lp(a), nmol/L	26.80 [10.00, 77.47]	27.70 [10.80, 78.97]	30.10 [10.70, 84.75]	26.50 [10.10, 77.85]	23.70 [8.50, 68.30]	< 0.001
Homocysteine, umol/L	13.40 [11.30, 16.50]	13.10 [11.10, 15.50]	13.30 [11.40, 16.80]	13.60 [11.50, 17.00]	13.60 [11.30, 17.00]	< 0.001
HbA1c, %	6.10 [5.70, 6.90]	5.80 [5.50, 6.20]	5.90 [5.60, 6.60]	6.20 [5.70, 7.10]	6.80 [6.00, 8.00]	< 0.001
hs‐TnI, pg/mL	4.10 [2.30, 8.50]	3.70 [2.10, 6.80]	3.90 [2.20, 8.10]	4.10 [2.30, 8.90]	4.80 [2.60, 11.10]	< 0.001
Myoglobin, ng/mL	22.20 [17.20, 29.50]	22.70 [18.00, 29.30]	22.70 [17.50, 30.20]	21.80 [17.00, 29.00]	21.85 [16.48, 29.50]	0.003
CK‐MB, ng/mL	1.60 [1.10, 2.30]	1.60 [1.10, 2.30]	1.70 [1.10, 2.30]	1.60 [1.20, 2.30]	1.70 [1.20, 2.40]	0.358
Creatinine, µmol/L	74.90 [65.70, 85.30]	73.70 [64.70, 82.60]	74.40 [65.40, 84.40]	75.50 [66.27, 85.70]	76.60 [66.57, 89.30]	< 0.001
Urea, mmol/L	5.62 [4.71, 6.75]	5.40 [4.58, 6.44]	5.54 [4.65, 6.59]	5.63 [4.74, 6.82]	5.93 [5.00, 7.12]	< 0.001
Glucose, mmol/L	5.82 [5.04, 7.61]	5.14 [4.68, 5.72]	5.50 [4.96, 6.52]	6.13 [5.21, 7.80]	8.02 [6.11, 10.92]	< 0.001
Triglyceride, mmol/L	1.49 [1.09, 2.12]	0.98 [0.79, 1.19]	1.40 [1.12, 1.72]	1.74 [1.33, 2.23]	2.43 [1.78, 3.46]	< 0.001
TyG index	8.91 [8.50, 9.36]	8.33 [8.10, 8.53]	8.79 [8.57, 8.99]	9.13 [8.86, 9.37]	9.69 [9.31, 10.09]	< 0.001
hs‐CRP, mg/L	1.01 [0.55, 2.44]	0.48 [0.34, 0.71]	0.84 [0.55, 1.46]	1.43 [0.81, 2.83]	2.93 [1.30, 6.46]	< 0.001
Uric acid, µmol/L	331.60 [277.75, 393.65]	317.90 [263.17, 371.70]	328.90 [280.10, 390.40]	336.30 [284.30, 395.42]	347.20 [284.50, 418.65]	< 0.001
ALT, U/L	20.00 [14.00, 29.00]	19.00 [14.00, 27.00]	20.00 [14.00, 29.00]	21.00 [15.00, 30.00]	21.00 [14.00, 31.00]	< 0.001
AST, U/L	19.00 [15.00, 23.00]	19.00 [16.00, 23.00]	19.00 [16.00, 23.00]	19.00 [15.00, 23.00]	18.00 [15.00, 24.00]	0.094
Total protein, g/L	72.50 [68.80, 76.00]	71.45 [67.80, 75.00]	72.60 [69.00, 75.90]	72.60 [69.20, 76.30]	73.10 [69.60, 76.80]	< 0.001
Albumin, g/L	45.30 [43.00, 47.40]	45.20 [42.90, 47.50]	45.60 [43.10, 47.60]	45.30 [43.00, 47.40]	45.10 [42.90, 47.10]	0.003
TB, µmol/L	10.64 [8.01, 14.20]	11.64 [8.80, 15.45]	10.98 [8.27, 14.40]	10.47 [7.83, 13.70]	9.64 [7.20, 12.82]	< 0.001
DB, µmol/L	4.41 [3.42, 5.69]	4.92 [3.84, 6.34]	4.47 [3.53, 5.79]	4.33 [3.32, 5.45]	4.00 [3.13, 5.07]	< 0.001
TC, mmol/L	3.84 [3.26, 4.56]	3.52 [3.04, 4.11]	3.73 [3.22, 4.39]	3.93 [3.33, 4.68]	4.24 [3.61, 5.05]	< 0.001
HDL‐C, mmol/L	1.05 [0.91, 1.24]	1.19 [1.02, 1.39]	1.07 [0.93, 1.27]	1.02 [0.89, 1.19]	0.94 [0.82, 1.10]	< 0.001
LDL‐C, mmol/L	2.02 [1.57, 2.59]	1.81 [1.43, 2.27]	1.98 [1.56, 2.50]	2.11 [1.65, 2.71]	2.22 [1.73, 2.86]	< 0.001
Glucose‐lowering therapy, *n* (%)	3541 (38.8)	548 (24.0)	692 (30.3)	932 (40.8)	1369 (60.0)	< 0.001
Antihypertensive therapy, *n* (%)	3878 (42.5)	846 (37.0)	916 (40.1)	1021 (44.7)	1095 (48.0)	< 0.001
Lipid‐lowering therapy, *n* (%)	8179 (89.6)	2064 (90.4)	2038 (89.3)	2061 (90.2)	2016 (88.4)	0.107
ACS subtype, *n* (%)						< 0.001
Unstable angina	8603 (94.2)	2201 (96.4)	2175 (95.3)	2134 (93.4)	2093 (91.8)	
NSTEMI	410 (4.5)	73 (3.2)	81 (3.5)	117 (5.1)	139 (6.1)	
STEMI	118 (1.3)	10 (0.4)	27 (1.2)	33 (1.4)	48 (2.1)	

*Note*: 1. Continuous data are presented as median (25th percentile, 75th percentile), and categorical data are presented as *n* (%).

2. Glucose‐lowering therapy was defined as the use of any medication with blood glucose‐lowering effects. Antihypertensive therapy was defined as the use of any medication with blood pressure‐lowering effects. Lipid‐lowering therapy was defined as the use of any medication with lipid‐lowering effects.

3. CTI quartiles were defined as follows: Q1, < 8.460; Q2, 8.460 to < 9.006; Q3, 9.006 to < 9.606; and Q4, ≥ 9.606.

Abbreviations: ALT, alanine aminotransferase; AST, aspartate aminotransferase; BMI, body mass index; CK‐MB, creatine kinase‐MB; CTI, C‐reactive protein–triglyceride‐glucose index; DB, direct bilirubin; HbA1c, glycated hemoglobin A1c; HDL‐C, high‐density lipoprotein cholesterol; high‐sensitivity troponin I; hs‐CRP, high‐sensitivity C‐reactive protein; hs‐TnI, LDL‐C, low‐density lipoprotein cholesterol; Lp(a), lipoprotein(a); TB, total bilirubin; TC, total cholesterol; TyG index, triglyceride‐glucose index.

### 2.2 Association of the TyG Index, hs‐CRP, and CTI for MACCE

To examine the associations of the TyG index, hs‐CRP, and CTI with MACCE, Cox proportional hazards regression models were fitted (Table 2).

**TABLE 2:** Multivariable Cox regression analysis of the associations of the TyG index, hs‐CRP, and CTI with MACCE.

Indices	Groups	events	HR 1 (95% CI)	*p* value	HR 2 (95% CI)	*p* value	HR 3 (95% CI)	*p* value
**TyG index**	Continuous		1.236 (1.121, 1.363)	< 0.001	1.263 (1.144, 1.393)	< 0.001	1.2347 (1.1153, 1.367)	< 0.001
	Q1 < 8.504	152	Reference		Reference		Reference	
	Q2 (8.504, 8.907)	204	1.36 (1.102, 1.678)	0.004	1.365 (1.107, 1.685)	0.004	1.341 (1.085, 1.658)	0.007
	Q3 (8.907, 9.362)	207	1.371 (1.112, 1.691)	0.003	1.385 (1.124, 1.708)	0.002	1.34 (1.083, 1.659)	0.007
	Q4 ≥ 9.362	238	1.58 (1.289, 1.936)	< 0.001	1.624 (1.324, 1.991)	< 0.001	1.553 (1.260, 1.915)	< 0.001
**hs‐CRP**	Continuous		1.039 (1.023, 1.055)	< 0.001	1.038 (1.022, 1.054)	< 0.001	1.031 (1.018, 1.047)	< 0.001
	Q1 < 0.56	166	Reference		Reference		Reference	
	Q2 (0.56, 1.02)	192	1.202 (0.977, 1.48)	0.082	1.213 (0.986, 1.493)	0.068	1.1804 (0.957, 1.455)	0.121
	Q3 (1.02, 2.45)	193	1.202 (0.977, 1.479)	0.082	1.211 (0.984, 1.49)	0.071	1.151 (0.932, 1.422)	0.193
	Q4 ≥ 2.45	250	1.569 (1.289, 1.909)	< 0.001	1.575 (1.294, 1.917)	< 0.001	1.43 (1.163, 1.757)	< 0.001
**CTI**	Continuous		1.267 (1.173, 1.369)	< 0.001	1.285 (1.189, 1.389)	< 0.001	1.248 (1.150, 1.355)	< 0.001
	Q1 < 8.460	146	Reference		Reference		Reference	
	Q2 (8.460, 9.006)	191	1.333 (1.075, 1.654)	0.009	1.336 (1.077, 1.658)	0.008	1.3068 (1.051, 1.625)	0.016
	Q3 (9.006, 9.606)	217	1.505 (1.22, 1.857)	< 0.001	1.524 (1.236, 1.88)	< 0.001	1.453 (1.172, 1.801)	< 0.001
	Q4 ≥ 9.606	247	1.736 (1.415, 2.13)	< 0.001	1.776 (1.447, 2.18)	< 0.001	1.651 (1.333, 2.045)	< 0.001

*Note*: Model 1: Unadjusted data.

Model 2: Minimally adjusted models were adjusted for age and sex.

Model 3: Multivariable adjusted models were additionally adjusted for body mass index, hypertension, homocysteine, albumin, total protein, aspartate aminotransferase, alanine aminotransferase, uric acid, lipid‐lowering therapy, antihypertensive medication, low‐density lipoprotein cholesterol, and creatinine.

Abbreviations: CTI, C‐reactive protein–triglyceride‐glucose index; CI, confidence interval; HR, hazard ratio; hs‐CRP, high‐sensitivity C‐reactive protein; MACCE, major adverse cardiovascular and cerebrovascular events; TyG index, triglyceride‐glucose index;.

When treated as continuous variables, the TyG index, hs‐CRP, and CTI were each significantly related to MACCE in the unadjusted Model 1. The corresponding hazard ratios (HRs) were 1.236 for the TyG index (95% CI: 1.121–1.363), 1.039 for hs‐CRP (95% CI: 1.023–1.055), and 1.267 for CTI (95% CI: 1.173–1.369), with all *p* < 0.001. Accounting for potential confounding factors did not attenuate these associations, which remained significant.

In Model 3 with full adjustment, every 1‐unit increase in the TyG index corresponded to a 23.5% higher hazard of MACCE (HR = 1.235, 95% CI: 1.115–1.367, *p* < 0.001), while each 1‐unit increment in hs‐CRP corresponded to a 3.1% higher hazard (HR = 1.031, 95% CI: 1.018–1.047, *p* < 0.001). CTI was also significantly related to MACCE risk in Model 3 (HR = 1.248, 95% CI: 1.150–1.355, *p* < 0.001).

In the analyses based on quartiles, MACCE risk generally increased across higher quartiles of the TyG index, hs‐CRP, and CTI. After adjustment for all covariates, patients in Q2, Q3, and Q4 of the TyG index had higher MACCE risks than those in Q1, with the largest effect estimate observed in Q4 (HR = 1.553, 95% CI: 1.260–1.915, *p* < 0.001). For hs‐CRP, Q4 corresponded to a 43.0% elevated risk of MACCE (HR = 1.430, 95% CI: 1.163–1.757, *p* < 0.001), whereas the associations for Q2 and Q3 were not statistically significant. Using Q1 as the reference category, MACCE risk rose progressively through CTI quartiles. The HRs for Q2, Q3, and Q4 were 1.307, 1.453, and 1.651, respectively, and all associations reached statistical significance (all *p* < 0.05). The largest estimate was seen in Q4 (HR = 1.651, 95% CI: 1.333–2.045, *p* < 0.001). In addition, pairwise interaction analyses of the TyG index, homocysteine (HCY), and uric acid (UA) are summarized in Table S3.

### 2.3 Predictive Performance of hs‐CRP, the TyG Index, and CTI

We used receiver operating characteristic analysis to assess differences in the discriminatory performance of hs‐CRP, the TyG index, and CTI for MACCE risk, as shown in Figure 2 and Table 3. The AUC for the basic model was 0.5809 (95% CI: 0.5601–0.6016). Adding hs‐CRP, the TyG index, and CTI increased the AUC to 0.5857, 0.5938, and 0.5983, respectively. Among the three biomarkers, CTI showed the highest AUC, although the absolute improvement over the basic model was modest. Compared with the basic model, the CTI model significantly improved the net reclassification improvement (NRI), whereas the improvement in the integrated discrimination improvement (IDI) was not statistically significant.

**FIGURE 2:**
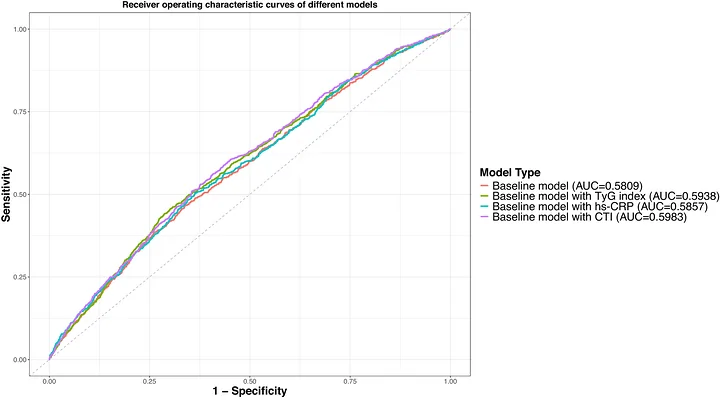
Receiver operating characteristic curves comparing the predictive performance of the basic model and models also including hs‐CRP, the TyG index, or CTI.

**TABLE 3:** Predictive performance of models with hs‐CRP, the TyG index, and CTI for MACCE.

Model	AUC (95% CI)	*p* value	IDI (95% CI)	*p* value	NRI (95%CI)	*p* value
Basic model	0.5809 [0.5601, 0.6016]					
Basic model + hs‐CRP	0.5857 [0.565, 0.6065]	0.2052	0.0035 [−0.0016, 0.0096]	0.214	0.0022 [0.001, 0.0034]	< 0.001
Basic model + TyG index	0.5938 [0.5732, 0.6143]	0.0169	0.0006 [−0.0011, 0.0034]	0.752	0.0020 [0.001, 0.0029]	< 0.001
Basic model + CTI	0.5983 [0.5777, 0.6183]	0.0070	0.0038 [−0.0005, 0.0089]	0.100	0.0036 [0.0023, 0.0049]	< 0.001

*Note*: The basic model included the same covariates as Model 3 in Table 2. The *p* values for AUC were calculated by comparing each expanded model with the basic model. IDI and NRI were calculated to evaluate the incremental predictive value of adding each biomarker to the basic model.

Abbreviations: AUC, area under the receiver operating characteristic curve; CI, confidence interval; CTI, C‐reactive protein–triglyceride‐glucose index; IDI, integrated discrimination improvement; MACCE, major adverse cardiovascular and cerebrovascular events; NRI, net reclassification improvement; TyG, triglyceride‐glucose.

### 2.4 Restricted Cubic Spline Analyses and Kaplan–Meier Curves for Associations With MACCE Risk

Restricted cubic spline (RCS) analyses showed that higher levels of the TyG index, hs‐CRP, and CTI corresponded to greater MACCE risk, without statistically significant evidence of nonlinearity (all *p* for nonlinearity > 0.05; Figure 3). Overall, MACCE risk increased progressively with increasing levels of these indices. Unadjusted RCS analyses showed similar positive associations, with no significant evidence of nonlinearity, as shown in Figure S1.

**FIGURE 3:**
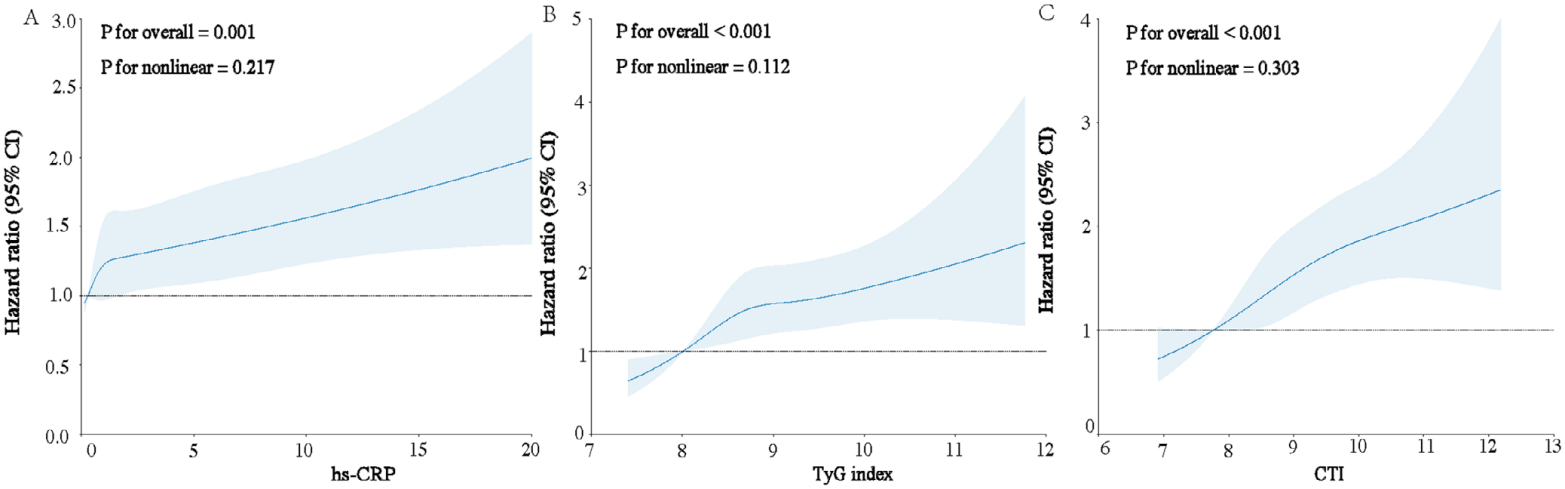
Restricted cubic spline analyses of the associations of hs‐CRP, the TyG index, and CTI with MACCE risk.

To better characterize the correlation between CTI quartiles and MACCE events, we allocated patients into quartile‐based groups (Q1–Q4) based on their CTI quartile and generated Kaplan–Meier cumulative incidence curves. As illustrated in Figure 4, cumulative MACCE incidence generally increased across CTI quartiles, the highest event burden being observed in Q4. The log‐rank test demonstrated significant differences in cumulative MACCE incidence among the four CTI quartiles (*p* < 0.001).

**FIGURE 4:**
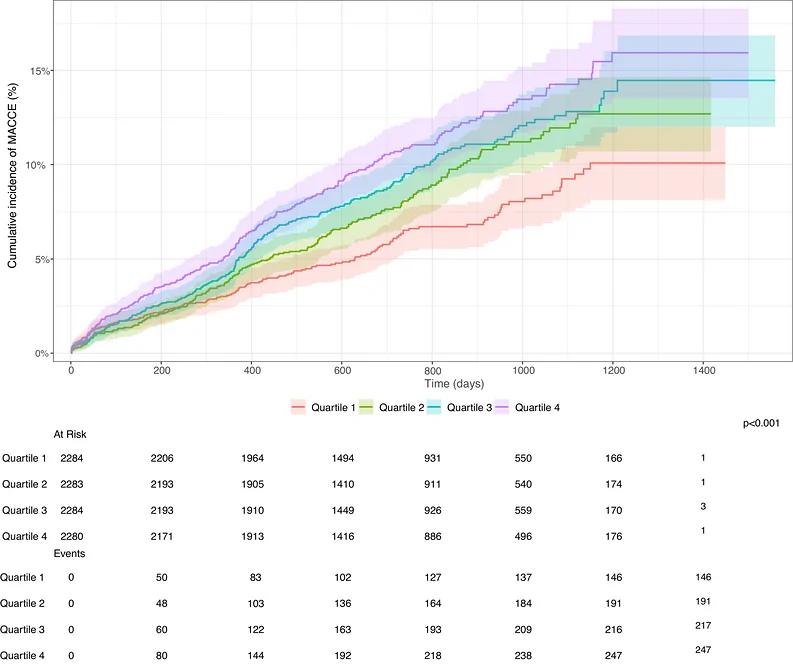
Kaplan–Meier cumulative incidence curves for MACCE according to CTI quartiles.

### 2.5 Subgroup Analysis of the Association Between CTI and MACCE

Subgroup analyses examined whether the relationship between CTI and MACCE risk was consistent across different patient groups. As illustrated in Figure 5, higher CTI levels corresponded to greater MACCE risk within subgroups defined by age, sex, BMI, hypertension, and diabetes status. The association remained statistically significant within each subgroup. Interaction analyses revealed no significant heterogeneity across these subgroups, with all interaction *p* values > 0.05, a result demonstrating the general consistency of the CTI–MACCE relationship across different clinical populations.

**FIGURE 5:**
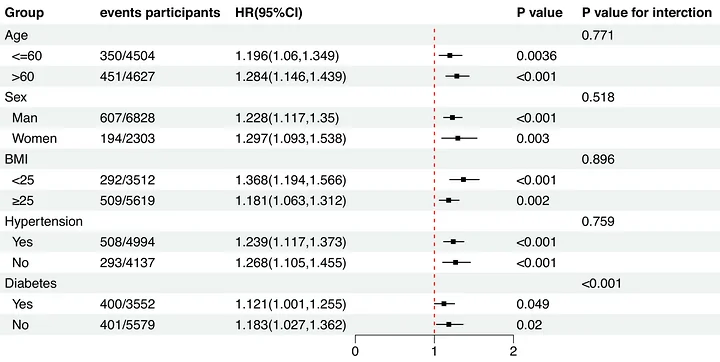
Subgroup analysis of the association between CTI and MACCE.

### 2.6 Exploratory Joint Association of the TyG Index and hs‐CRP With MACCE Risk

The exploratory joint relationship of the TyG index and hs‐CRP to MACCE risk was further evaluated, with the results summarized in Table S4. High and low levels were defined according to the respective median values. In the joint analysis, the low hs‐CRP/low TyG index category served as the reference. Isolated elevation of hs‐CRP showed no statistically significant association with MACCE risk (HR = 1.202, 95% CI: 0.973–1.486, *p* = 0.088), whereas isolated elevation of the TyG index showed a significant association with greater MACCE risk (HR = 1.261, 95% CI: 1.023–1.554, *p* = 0.030). The highest MACCE risk was identified among patients with elevated levels of both hs‐CRP and the TyG index (HR = 1.404, 95% CI: 1.153–1.708, *p* < 0.001).

## 3 Discussion

This large Chinese cohort of patients with ACS allowed us to examine the prognostic relevance of CTI for long‐term MACCE. To our knowledge, evidence regarding the prognostic value of CTI for long‐term MACCE in broad ACS populations remains limited. Following adjustment for multiple covariates, CTI was still independently related to MACCE risk. The AUC was numerically higher for CTI than for either the TyG index or hs‐CRP used separately, although the absolute improvement was modest. The significant improvement in NRI suggests potential incremental value for risk reclassification, whereas the non‐significant IDI and modest increase in AUC indicate that the incremental predictive utility of CTI should be interpreted cautiously. In exploratory joint analyses, individuals with an elevated TyG index and high hs‐CRP showed the highest MACCE risk, suggesting that the joint burden of metabolic disturbance and systemic inflammation may be associated with an adverse prognosis. In addition, the relationship between CTI and MACCE risk remained largely similar across major clinical subgroups, as defined by age, sex, BMI, hypertension, and diabetes status.

This study has several potential methodological and clinical implications. First, compared with biomarkers that reflect metabolic disturbances or inflammation alone, CTI integrates TyG‐related metabolic information with C‐reactive protein‐related inflammatory information into a single index, providing a biologically relevant measure of the metabolic and inflammatory processes involved in ACS progression. Second, based on a large real‐world cohort of 9131 patients with ACS, our study provides additional evidence of an association between CTI and long‐term MACCE risk, extending previous findings that have mainly focused on short‐term outcomes or selected populations undergoing percutaneous coronary intervention (PCI). Third, the relationship between CTI and MACCE showed a broadly similar pattern across major clinical subgroups, suggesting that CTI may have potential applicability across diverse ACS patient profiles. From a clinical perspective, CTI is calculated using routinely available laboratory parameters, making it a practical adjunct to post‐ACS risk assessment. Patients with elevated CTI may represent a subgroup with concomitant metabolic and inflammatory abnormalities, for whom closer follow‐up and comprehensive risk‐factor management may be warranted.

The present results broaden the potential value of CTI for long‐term prognostic risk assessment in a broad ACS population. As a composite biomarker, CTI integrates two key pathophysiological dimensions—metabolism and inflammation. It reflects insulin resistance‐related metabolic status through the TyG index and systemic inflammation through hs‐CRP [19]. This composite index is biologically plausible in the context of the metabolic‐inflammatory interactions involved in ACS progression [24]. Experimental studies suggest that IR may stimulate NADPH oxidase activity and enhance the production of reactive oxygen species, induce mitochondrial dysfunction, and promote endothelial cell apoptosis, thereby accelerating atherosclerosis progression [25]. Meanwhile, chronic inflammation can further exacerbate endothelial dysfunction by enhancing endothelial pro‐adhesive signaling, promoting the recruitment of circulating inflammatory cells into the vascular wall, and disrupting vascular barrier integrity [26]. Moreover, cytokines with pro‐inflammatory effects, including TNF‐α and IL‐6, may interfere with insulin signaling pathways and exacerbate metabolic disturbances, potentially contributing to a vicious cycle of metabolic dysfunction and inflammation [27, 28].

Previous investigations have separately evaluated the relationships between the TyG index and hs‐CRP with ACS prognosis. A meta‐analysis by Liang et al. demonstrated that a higher TyG index was linked to a greater likelihood of coronary heart disease, greater disease severity, and poorer clinical outcomes [29]. Wang et al. reported that an elevated TyG index independently predicted subsequent MACCE in patients with ACS and that its inclusion improved the predictive performance of risk models [13]. Dong et al. similarly reported that among patients with ACS and a history of undergoing coronary artery bypass grafting who underwent PCI, a higher TyG index independently predicted MACCE [30]. Beyond metabolic dysfunction, inflammatory activation is closely associated with plaque progression, plaque destabilization, and unfavorable cardiovascular events. Lawler et al. highlighted hs‐CRP as a clinically useful biomarker for assessing vascular inflammation and cardiovascular risk [17]. Ridker et al. further emphasized IL‐6 signaling as an important inflammatory pathway in cardiovascular disease, with CRP serving primarily as a downstream inflammatory biomarker [18].

CTI showed a consistent association with MACCE in both continuous and quartile‐based analyses. In addition, CTI demonstrated an AUC numerically greater than that obtained with hs‐CRP and the TyG index, though the absolute improvement was modest. From a clinical perspective, elevated CTI may identify a subgroup of patients with ACS who have a greater combined metabolic and inflammatory burden and may offer supplementary information to support risk stratification. The potential value of CTI compared with the TyG index or hs‐CRP alone may be attributable to its integrated nature. By simultaneously incorporating information on IR and systemic inflammation, CTI may provide a more comprehensive representation of the underlying pathophysiological state. Moreover, atherosclerosis is closely related to the interplay between metabolic disturbances and chronic inflammation, and previous studies have also supported the relevance of this combined exposure pattern [24]. In addition, a single biomarker may capture only one aspect of disease pathophysiology, whereas a composite index can integrate information from multiple related pathways and provide additional prognostic information.

In the present study, elevated CTI levels showed an association with an increased risk of MACCE among individuals with ACS. RCS analyses indicated a positive association between CTI and MACCE risk, without significant evidence of nonlinearity, suggesting that MACCE risk generally increased with increasing CTI levels and that no distinct threshold effect was observed within this cohort. Analysis within subgroups showed that the correlation between CTI and MACCE remained broadly similar across the subgroups defined by age, sex, BMI, hypertension, and diabetes, with no interaction reaching significance. These results indicate that CTI may provide consistent prognostic information across different clinical profiles of patients with ACS. However, the clinical utility of CTI for guiding individualized risk management requires further validation in independent cohorts.

The pathophysiological role of CTI in ACS remains incompletely understood; however, our joint evaluation of both the TyG index and hs‐CRP provides additional insight into the potential interplay between metabolic dysfunction and systemic inflammation. MACCE risk was highest among patients with both an elevated TyG index and elevated hs‐CRP, suggesting that the coexistence of inflammatory and metabolic abnormalities may be linked to a less favorable prognosis. This finding is consistent with a metabolic‐inflammatory framework in which metabolic dysfunction and inflammation may reinforce one another, thereby contributing to atherosclerotic progression and plaque instability. Thus, CTI may serve as an integrated marker reflecting the combined burden of these two interrelated pathological processes.

The present study is subject to a number of limitations. First, CTI was derived from a single set of baseline measurements of hs‐CRP, triglycerides, and glucose. Therefore, the potential impact of dynamic changes in these biomarkers over time on MACCE risk could not be assessed. In addition, hs‐CRP measured during ACS hospitalization may be influenced by acute‐phase inflammatory responses; thus, CTI should be interpreted as a composite metabolic‐inflammatory index rather than a marker of chronic inflammation alone. Second, as an observational study, although multiple potential confounders were adjusted for in the multivariable models, residual effects of unmeasured factors, including socioeconomic status and medication adherence, cannot be completely excluded. Third, all participants were recruited from a single hospital, and most were middle‐aged or older Chinese patients with ACS. Therefore, the representativeness and generalizability of our findings require further validation across different regions, ethnic groups, and broader clinical settings.

## 4 Conclusion

Overall, higher levels of CTI, the TyG index, and hs‐CRP corresponded to a greater risk of long‐term MACCE among patients with ACS. CTI demonstrated numerically higher predictive performance than either the TyG index or hs‐CRP alone and may provide additional prognostic information, although the incremental improvement was modest. The present findings suggest that CTI could be used as a readily available adjunctive metabolic‐inflammatory marker for risk assessment in patients with ACS. Further studies are needed to establish its clinical utility in independent, multicenter cohorts.

## 5 Materials and Methods

### 5.1 Study Population

In this prospective observational investigation at a single center, we consecutively enrolled 12,170 patients with confirmed ACS at Beijing Anzhen Hospital between 2021 and 2024. Of these, 1124 patients were excluded due to loss of follow‐up. Another 1915 lacked fasting glucose, triglyceride, or hs‐CRP values and were not included in the analysis. The final analytic cohort comprised 9131 patients, as depicted in Figure 1.

### 5.2 Calculation of CTI and TyG

hs‐CRP and the TyG index were applied as indicators of inflammatory condition and IR, respectively. The CTI incorporates both hs‐CRP and the TyG index in its calculation. The calculation formulas for CTI and the TyG index were as follows:

CTI = 0.412 × ln[hs‐CRP (mg/L)] + ln [TG (mg/dL) × glucose (mg/dL)/2] [19];

TyG index = ln [triglycerides (mg/dL) × glucose (mg/dL)/2] [31].

### 5.3 Data Collection and Definitions

Trained study staff gathered baseline information using standardized questionnaires and clinical records, including the following: (1) Demographic characteristics: sex and age; (2) Anthropometric measurements: BMI as the primary indicator of obesity; (3) Clinical history and medication use: previous diagnoses of hypertension and diabetes, as well as current medication use; and (4) Laboratory measurements: lipoprotein(a) [Lp(a)], glycated hemoglobin (HbA1c), high‐sensitivity troponin I (hs‐TnI), creatine kinase‐MB (CK‐MB), glucose, triglycerides (TG), HDL‐C, LDL‐C, hs‐CRP, TC, total bilirubin (TB), direct bilirubin (DB), Cr, urea, HCY, and UA. The diagnosis of hypertension was based on a prior physician diagnosis of the condition, SBP ≥ 140 mmHg and/or DBP ≥ 90 mmHg, or ongoing antihypertensive therapy [32]. Diabetes mellitus (DM) was defined by a prior physician diagnosis of diabetes, fasting plasma glucose ≥ 7.0 mmol/L, 2‐h plasma glucose during an oral glucose tolerance test ≥ 11.1 mmol/L, HbA1c ≥ 6.5%, or current treatment with antidiabetic medication [33]. ACS referred to unstable angina pectoris (UAP), non‐ST‐segment elevation myocardial infarction (NSTEMI), and ST‐segment elevation myocardial infarction (STEMI), diagnosed in accordance with the Fourth Universal Definition of Myocardial Infarction (MI) and the ESC Guidelines for the management of acute coronary syndromes [7, 34, 35].

### 5.4 Endpoints

The primary outcome was MACCE, defined as a composite encompassing all‐cause death, nonfatal myocardial infarction, nonfatal stroke, and unplanned revascularization. Death from any cause, whether cardiovascular or non‐cardiovascular, was classified as all‐cause death. The secondary outcomes comprised all‐cause death, nonfatal MI, and nonfatal stroke. Follow‐up commenced on the date of hospital admission. Outcome events were determined through standardized telephone follow‐up after discharge. Among patients experiencing MACCE, the follow‐up interval extended from hospital admission to the date of the first occurrence of MACCE. For patients who did not experience MACCE, follow‐up was censored on the last follow‐up date or August 31, 2024, whichever came first. Patients were followed for a median of 707 days. Outcome events were adjudicated by trained investigators who were blinded to biomarker data.

### 5.5 Statistical Analysis

Participants who lacked data on hs‐CRP, glucose, or triglycerides were excluded because calculating CTI and the TyG index necessitated these variables. For other baseline covariates, missing observations were imputed using the random forest method via the R package mice. Assuming a missing‐at‐random mechanism, five datasets were created through imputation. Data for continuous variables were summarized as mean ± SD or as median with interquartile range, and analysis of variance or the Kruskal–Wallis test assessed differences between groups. Data for categorical variables were expressed as frequencies and percentages, and comparisons were made using the chi‐square or Fisher's exact test. Baseline profiles were compared across the four CTI quartiles.

Cox proportional hazards models were constructed to derive HRs and 95% CIs for the associations of CTI, the TyG index, and hs‐CRP with MACCE. Each biomarker was analyzed as a continuous variable and by quartile, using Q1 as the reference. Model 1 was unadjusted, Model 2 included age and sex, and Model 3 further included BMI, hypertension, HCY, albumin, total protein, aspartate aminotransferase, alanine aminotransferase, lipid‐lowering therapy, antihypertensive medication use, LDL‐C, Cr, and UA. Cumulative MACCE incidence across CTI quartiles was estimated with Kaplan–Meier curves, with between‐group differences assessed by the log‐rank test. RCS analyses were used to characterize the trends in the relationships of CTI, the TyG index, and hs‐CRP with MACCE risk and to examine nonlinearity, with adjustment for the same covariates as in Model 3. The AUC, IDI, and NRI were calculated to assess the incremental prognostic contribution of CTI. To investigate how the TyG index and hs‐CRP jointly relate to MACCE risk, we performed a joint analysis (Table S4). In addition, subgroup analyses used strata defined by age, sex, BMI, hypertension, and diabetes, and corresponding interaction terms were included to assess whether these characteristics modified the association between CTI and MACCE. In our study, R software (version 4.1.3; Vienna, Austria) and IBM SPSS Statistics (version 26.0; IBM Corp., Armonk, NY, USA) were used for statistical analyses. Two‐tailed tests were used throughout, with statistical significance defined as *p* < 0.05.

## Author Contributions

Wenjie Wang and Xiangfei Meng designed the study, analyzed and interpreted the data, and drafted the original manuscript. Yang Zhang, Li Lin, Zehao Zhao, Jiayi Han, Ruixiang Feng, Yingxuan Bai, and Liwei Guo recruited the subjects, performed patient assessments, and critically reviewed the manuscript for intellectual content. Yong Zeng and Yaodong Ding supervised the study, interpreted data, and made critical revisions to the paper. Dr. Yong Zeng is the guarantor of this work, and had full access to all the data in the study, and takes responsibility for the integrity of the data and the accuracy of the data analysis. All authors have read and approved the final manuscript.

## Funding

This work was supported by the National Natural Science Foundation of China (grant no.: 82270345), the Beijing Municipal Healthcare Administration (grant no.: YGLX202324), the First‐Launch Project of Beijing Municipal Health Commission (grant no.: 2024‐1‐2061), and Beijing Anzhen Hospital High ‐ Level Research Special Project (grant no.: 2024AZB1011).

## Ethics Statement

The study was approved by the Ethics Committee of Beijing Anzhen Hospital, Capital Medical University (approval No. KS2023081). Written informed consent was obtained from all the participants prior to enrollment. All the methods were performed in accordance with the Declaration of Helsinki and relevant guidelines and regulations.

## Conflicts of Interest

The authors declare no conflicts of interest.

## Supporting information




**Supplementary Table S1**: Follow‐up outcomes according to CTI quartiles.
**Supplementary Table S2**: Pairwise interaction analyses among TyG index, homocysteine, and uric acid for MACCE risk.
**Supplementary Table S3**: Baseline characteristics of patients with and without MACCE.
**Supplementary Table S4**: Joint association of the TyG index and hs‐CRP with MACCE risk.
**Supplementary Figure S1**: Unadjusted restricted cubic spline analyses of the associations of hs‐CRP, the TyG index and CTI with MACCE risk.

## Data Availability

The datasets generated and analyzed during the current study are available from the corresponding author on reasonable request.
