# Supplementary material for: Comparative Prognostic Value of the Triglyceride‐Glucose Index, hs‐CRP, and the Derived C‐Reactive Protein–Triglyceride‐Glucose Index for Major Adverse Cardiovascular and Cerebrovascular Events in Acute Coronary Syndrome: Insights From a Prospective Cohort
Source: MedComm (2020). 2026 Nov 13;7(11):e71130. doi: 10.1002/mco2.71130 (PMC13761297; doi:10.1002/mco2.71130)
Supplement: Supplementary file 1 [file MCO2-7-e71130-s001.docx]

Comparative prognostic value of triglyceride-glucose index, hs-CRP, and their derived C-reactive protein-triglyceride glucose index for major adverse cardiovascular and cerebrovascular events in acute coronary syndrome: insights from a prospective cohort

Wenjie Wang1#, Xiangfei Meng1#, Yang Zhang1, Li Lin1, Zehao Zhao1, Jiayi Han1, Ruixiang Feng1, Yingxuan Bai1, Liwei Guo1, Yaodong Ding1*, Yong Zeng1*

^1^Department of Cardiology, Beijing Anzhen Hospital, Capital Medical University, Beijing, PR China

\# The authors contribute equally to this work.

*** Correspondence:**

**Yong Zeng: yzeng_anzhen@mail.ccmu.edu.cn;**

**Yaodong Ding: [dingyaodong1@163.com;](mailto:dingyaodong1@163.com;)**

Phone Number: +86 81992150

Supplementary Table S1. Follow-up outcomes according to CTI quartiles.

| **Outcomes** | **Overall**  **(n=9,131)** | **Quartile 1**  **(＜8.460; n=2,284)** | **Quartile 2**  **(8.460 - 9.006**;**n=2,283)** | **Quartile 3**  **(9.006 - 9.606, n=2,284)** | **Quartile 4**  **(≥9.606, n=2,280)** | **P value** |
| --- | --- | --- | --- | --- | --- | --- |
| Death, n (%) | 118 ( 1.3) | 21 ( 0.9) | 37 ( 1.6) | 27 ( 1.2) | 33 ( 1.4) | 0.167 |
| Myocardial infarction, n (%) | 181 ( 2.0) | 30 ( 1.3) | 50 ( 2.2) | 44 ( 1.9) | 57 ( 2.5) | 0.030 |
| Unplanned revascularization,  n (%) | 595 ( 6.5) | 113 ( 4.9) | 140 ( 6.1) | 161 ( 7.0) | 181 (7.9) | <0.001 |
| Stroke, n(%) | 192 ( 2.1) | 29 ( 1.3) | 49 ( 2.1) | 51 ( 2.2) | 63 ( 2.8) | 0.005 |
| MACCE, n (%) | 801 ( 8.8) | 146 ( 6.4) | 191 ( 8.4) | 217 ( 9.5) | 247 (10.8) | <0.001 |

^a.^ Data are presented as n (%).

^b.^ MACCE was defined as the occurrence of all-cause death, nonfatal myocardial infarction, nonfatal stroke, or unplanned revascularization during follow-up.

^c.^ Abbreviations: CTI, C-reactive protein–triglyceride-glucose index; MACCE, major adverse cardiovascular and cerebrovascular events.

^d.^ CTI quartiles were defined as follows: Q1, <8.460; Q2, 8.460 to <9.006; Q3, 9.006 to <9.606; and Q4, ≥9.606.

^e.^Component outcomes were recorded separately and were not mutually exclusive; therefore, the sum of component events may exceed the total number of MACCE events.

Supplementary Table S2. Pairwise interaction analyses among TyG index, homocysteine, and uric acid for MACCE risk.

| **Interaction term** | **HR for interaction** | **95% CI** | **P for interaction** |
| --- | --- | --- | --- |
| TyG index × Homocysteine | 0.999 | 0.989 - 1.010 | 0.902 |
| TyG index × Uric acid | 0.999 | 0.998 - 1.000 | 0.090 |
| Homocysteine × Uric acid | 1.00007 | 1.00002 - 1.00013 | 0.006 |

^a.^ Data are presented as hazard ratios (HRs) and 95% confidence intervals (CIs) for the interaction terms derived from Cox proportional hazards regression models. Each model included the corresponding two main-effect variables and their multiplicative interaction term. A two-sided P value <0.05 was considered statistically significant.

^b.^ TyG, triglyceride-glucose; MACCE, major adverse cardiovascular and cerebrovascular events; CI, confidence interval; HR, hazard ratio.

Supplementary Table S3. Baseline characteristics of patients with and without MACCE.

| **Characteristics** | **Overall**  **(n= 9,131)** | **Non - MACCE**  **( n=8,330)** | **MACCE**  **(n=801)** | **P value** |
| --- | --- | --- | --- | --- |
| Age, years | 61.00 [54.00, 67.00] | 61.00 [54.00, 67.00] | 62.00 [55.00, 68.00] | <0.001 |
| Male, n (%) | 6828 (74.8) | 6221 ( 74.7) | 607 ( 75.8) | 0.526 |
| BMI, kg/m² | 25.91 [23.89, 28.06] | 25.88 [23.89, 28.04] | 26.03 [24.04, 28.31] | 0.148 |
| Hypertension, n(%) | 4994 (54.7) | 4486 ( 53.9) | 508 ( 63.4) | <0.001 |
| Lp(a), nmol/L | 26.80 [10.00, 77.47] | 26.60 [10.00, 76.57] | 28.30 [10.30, 87.82] | 0.224 |
| Homocysteine,  umol/L | 13.40 [11.30, 16.50] | 13.30 [11.30, 16.40] | 13.90 [11.60, 18.20] | <0.001 |
| HbA1c,% | 6.10 [5.70, 6.90] | 6.00 [5.60, 6.90] | 6.30 [5.70, 7.50] | <0.001 |
| hs-TnI, pg/mL | 4.10 [2.30, 8.50] | 4.00 [2.30, 8.30] | 4.90 [2.60, 11.70] | <0.001 |
| Myoglobin, ng/mL | 22.20 [17.20, 29.50] | 22.15 [17.10, 29.40] | 22.90 [17.85, 31.05] | 0.049 |
| CK-MB, ng/mL | 1.60 [1.10, 2.30] | 1.60 [1.10, 2.30] | 1.70 [1.20, 2.40] | 0.269 |
| Creatinine, μmol/L | 74.90 [65.70, 85.30] | 74.80 [65.60, 85.10] | 76.60 [67.40, 86.80] | 0.001 |
| Urea, mmol/L | 5.62 [4.71, 6.75] | 5.60 [4.70, 6.72] | 5.87 [4.85, 7.03] | <0.001 |
| Glucose, mmol/L | 5.82 [5.04, 7.61] | 5.79 [5.04, 7.54] | 6.18 [5.15, 8.23] | <0.001 |
| Triglyceride, mmol/L | 1.49 [1.09, 2.12] | 1.49 [1.08, 2.12] | 1.55 [1.14, 2.17] | 0.008 |
| TyG index | 8.91 [8.50, 9.36] | 8.90 [8.49, 9.35] | 9.00 [8.61, 9.44] | <0.001 |
| hs-CRP, mg/L | 1.01 [0.55, 2.44] | 1.00 [0.54, 2.36] | 1.23 [0.62, 3.13] | <0.001 |
| CTI | 9.06 (0.85) | 9.04 (0.85) | 9.24 (0.85) | <0.001 |
| Uric acid, μmol/L | 339.53 (89.81) | 339.24 (89.30) | 342.45 (94.98) | 0.335 |
| ALT, U/L | 20.00 [14.00, 29.00] | 20.00 [14.00, 29.00] | 19.00 [13.00, 28.00] | 0.051 |
| AST, U/L | 19.00 [15.00, 23.00] | 19.00 [15.00, 23.00] | 18.00 [15.00, 24.00] | 0.015 |
| Total protein, g/L | 72.50 [68.80, 76.00] | 72.50 [68.90, 76.00] | 72.40 [68.70, 76.10] | 0.818 |
| Albumin, g/L | 45.30 [43.00, 47.40] | 45.30 [43.00, 47.40] | 45.00 [42.70, 47.30] | 0.029 |
| TB, μmol/L | 10.64 [8.01, 14.20] | 10.68 [8.04, 14.24] | 10.18 [7.65, 13.61] | 0.003 |
| TC, mmol/L | 3.84 [3.26, 4.56] | 3.84 [3.26, 4.55] | 3.90 [3.33, 4.67] | 0.030 |
| HDL- C, mmol/L | 1.05 [0.91, 1.24] | 1.05 [0.91, 1.25] | 1.02 [0.88, 1.19] | <0.001 |
| LDL- C, mmol/L | 2.02 [1.57, 2.59] | 2.01 [1.56, 2.58] | 2.08 [1.62, 2.67] | 0.049 |
| Glucose-lowering therapy, n(%) | 3541 (38.8) | 3143 ( 37.7) | 398 ( 49.7) | <0.001 |
| Antihypertensive therapy, n(%) | 3878 (42.5) | 3483 ( 41.8) | 395 ( 49.3) | <0.001 |
| Lipid-lowering therapy, n(%) | 8179 (89.6) | 7452 ( 89.5) | 727 ( 90.8) | 0.275 |

^a.^Data are presented as median [25th percentile, 75th percentile], mean (standard deviation), or n (%), as appropriate. P values were calculated for comparisons between patients with and without MACCE.

^b.^ MACCE, major adverse cardiovascular and cerebrovascular events; BMI, body mass index; TyG, triglyceride-glucose; hs-CRP, high-sensitivity C-reactive protein; ALT, alanine aminotransferase; AST, aspartate aminotransferase; HDL-C, high-density lipoprotein cholesterol; LDL-C, low-density lipoprotein cholesterol; HbA1c, glycated hemoglobin A1c; hs-TnI, high-sensitivity troponin I; CK-MB, creatine kinase-MB; Lp(a), lipoprotein(a); CTI, C-reactive protein–triglyceride-glucose index.

Supplementary Table S4. Joint association of TyG index and hs-CRP with MACCE risk.

| **Indices** | **Model 1 HR (95% CI)** | **P value** | **Model 2 HR (95% CI)** | **P value** |
| --- | --- | --- | --- | --- |
| **Scenario 1** |  |  |  |  |
| hs-CRP <median |  |  |  |  |
| TyG |  |  |  |  |
| Q1 | Reference |  | Reference |  |
| Q2 | 1.564(1.164,2.099) | 0.003 | 1.552(1.151,2.093) | 0.004 |
| Q3 | 1.48(1.09,2.01) | 0.012 | 1.4645(1.07,2.004) | 0.017 |
| Q4 | 1.742(1.284,2.363) | <0.001 | 1.7491(1.278,2.395) | <0.001 |
| hs-CRP ≥ median |  |  |  |  |
| TyG |  |  |  |  |
| Q1 | Reference |  | Reference |  |
| Q2 | 1.133(0.84,1.528) | 0.414 | 1.135(0.839,1.535) | 0.411 |
| Q3 | 1.189(0.891,1.588) | 0.239 | 1.17(0.872,1.57) | 0.295 |
| Q4 | 1.329(1.007,1.754) | 0.045 | 1.316(0.99,1.749) | 0.059 |
| **Scenario 2** |  |  |  |  |
| TyG < median |  |  |  |  |
| hs-CRP |  |  |  |  |
| Q1 | Reference |  | Reference |  |
| Q2 | 1.286(0.961,1.721) | 0.091 | 1.254(0.934,1.683) | 0.133 |
| Q3 | 1.132(0.826,1.552) | 0.44 | 1.081(0.784,1.49) | 0.637 |
| Q4 | 1.709(1.292,2.262) | <0.001 | 1.623(1.209,2.179) | 0.001 |
| TyG ≥ median |  |  |  |  |
| hs-CRP |  |  |  |  |
| Q1 | Reference |  | Reference |  |
| Q2 | 1.059(0.786,1.426) | 0.708 | 1.061(0.786,1.432) | 0.698 |
| Q3 | 1.123(0.844,1.495) | 0.425 | 1.084(0.81,1.451) | 0.587 |
| Q4 | 1.348(1.02,1.782) | 0.036 | 1.219(0.91,1.634) | 0.184 |
| **Scenario 3** |  |  |  |  |
| hs-CRP <median &TyG < median | Reference |  | Reference |  |
| hs-CRP ≥median &TyG <median | 1.271(1.032,1.565) | 0.024 | 1.202(0.973,1.486) | 0.088 |
| hs-CRP <median &TyG ≥ median | 1.261(1.025,1.551) | 0.028 | 1.261(1.023,1.554) | 0.03 |
| hs-CRP ≥median &TyG ≥ median | 1.504(1.246,1.816) | <0.001 | 1.404(1.153,1.708) | <0.001 |

^a.^Abbreviations: TyG index, triglyceride-glucose index; hs-CRP, high-sensitivity C-reactive protein; MACCE, major adverse cardiovascular and cerebrovascular events; HR, hazard ratio; CI, confidence interval.

^b.^ Model 1 was unadjusted. Model 2 was adjusted for age, sex, body mass index, hypertension, homocysteine, albumin, total protein, aspartate aminotransferase, alanine aminotransferase, uric acid, creatinine, low-density lipoprotein cholesterol, lipid-lowering therapy, antihypertensive therapy, and glucose-lowering therapy.

^c.^ TyG index quartiles were defined as follows: Q1, < 8.504; Q2, 8.504 to <8.907; Q3, 8.908 to <9.362; and Q4, ≥9.362. hs-CRP quartiles were defined as follows: Q1, <0.56; Q2, 0.56 to <1.02; Q3, 1.02 to <2.45; and Q4, ≥2.45.

Supplementary Figure S1 Unadjusted restricted cubic spline analyses of the associations of hs-CRP, TyG index and CTI with MACCE risk.

(A) hs-CRP; (B) TyG index; (C) CTI. Solid lines represent hazard ratios, and shaded areas represent 95% confidence intervals. HR, hazard ratio; CI, confidence interval; hs-CRP, high-sensitivity C-reactive protein; TyG index, triglyceride-glucose index; CTI, C-reactive protein–triglyceride glucose index; RCS, restricted cubic spline; MACCE, major adverse cardiovascular and cerebrovascular events.
